# A distal regulatory element regulates Wx gene expression and the amylose content in rice

**DOI:** 10.3389/fpls.2025.1694305

**Published:** 2025-10-08

**Authors:** Qingqing Yang, Fuhua Dong, Wenjie Zhu, Dongsheng Zhao

**Affiliations:** ^1^ Jiangsu Key Laboratory of Crop Genomics and Molecular Breeding/Zhongshan Biological Breeding Laboratory/Key Laboratory of Plant Functional Genomics of the Ministry of Education, Agricultural College of Yangzhou University, Yangzhou, China; ^2^ Jiangsu Co-Innovation Center for Modern Production Technology of Grain Crops/Jiangsu Key Laboratory of Crop Genetics and Physiology, Yangzhou University, Yangzhou, China

**Keywords:** rice, *Wx*, amylose content, cis-regulatory element, grain quality

## Abstract

Granule-bound starch synthase I (GBSSI) is encoded by the *Waxy* (*Wx*) gene, which is responsible for amylose biosynthesis and is an important factor determining rice cooking and eating quality in rice grains. Although some studies have edited the promoter of *Wx* to fine-tuning gene expression level and amylose content, few studies have focused on the distal allelic variations of *Wx*. In this study, we identified and confirmed one distal cis-regulatory element (CRE) related to the *Wx* gene. Furthermore, the *cre* mutants were generated in the Nipponbare background carrying the *Wx^b^* or *Wx^a^* allele. The amylose content of *Wx^b^-cre* mutant decreased by 21.43%–31.51% compared with that of wild-type rice with the *Wx^b^* allele. However, no significant difference in amylose content was observed between wild-type plants and *Wx^a^-cre* mutants which carried the *Wx^a^* allele. The altered mature mRNA transcript and protein level of *Wx* gene caused by CRE showed a high consistency with their amylose content. Meanwhile, lower gelatinization temperature and final viscosity were found in the *Wx^b^-cre* mutants, whereas the grain shape and chalkiness and major agronomic traits were not affected. Possibly, the *cre* mutant causes the transcriptional reprogramming of starch metabolism. Overall, we identified a distal CRE to finely regulate the *Wx* gene expression and amylose content, which provided a potentially useful allele for quality improvement in rice breeding.

## 1 Introduction

Gene expression is regulated at multiple levels in eukaryotes. Transcriptional regulation is a complex biological process that directly determines gene expression patterns and levels, influencing the phenotypes, stress tolerance/resistance, and productivity of plants (Cui et al., 2023). A key mechanism of gene transcriptional regulation is the recognition of cis-regulatory elements (CREs) by transcription factors, which controls transcription, and CREs are short DNA sequence motifs within the proximal genome region of a gene (Biłas et al., 2016). Variations in CREs play crucial roles in fine-tuning gene expression (Cui et al., 2023). Targeting the coding regions and CREs of genes through the clustered regularly interspaced short palindromic repeat (CRISPR)/Cas system has been well established, enabling the modulation of target gene expression and the improvement of plant traits (Saeed et al., 2022).

As one of the most important food crops in the world, rice provides energy sources for over half of the global population (Mohapatra and Sahu, 2022). The improvement of grain quality, especially cooking, taste, and appearance quality, is an important goal in rice breeding (Lau et al., 2015). Rice grain contains over 80% starch, including amylose and amylopectin. The amylose content (AC) is considered the key component controlling the eating and cooking quality of the rice endosperm, and the *Waxy* (*Wx*) gene has been identified to control AC (Li et al., 2016; Sano, 1984). The *Wx* gene encodes granule-bound starch synthase I (GBSSI), and at least nine natural *Wx* alleles have been identified in rice, causing differences in AC and rice quality (Teng et al., 2012; Zhang et al., 2021, 2019; Zhou et al., 2021). Due to specialized rice consumption, the demand for AC in rice is becoming increasingly diversified, which requires the regulation of AC or the generation of new *Wx* alleles.

Gene editing CRE is an improved method for fine-tuning *Wx* gene expression and AC compared with searching for natural or mutated alleles (Ding et al., 2021). There have been several successful reports of fine-tuning AC in the *Wx^b^* background by editing CREs in the promoter region of the *Wx* gene (Zhao et al., 2024). A previous study demonstrated that editing the promoter regulates *Wx* expression, changing AC in transgenic lines (Zeng et al., 2020; Huang et al., 2020). However, these studies have mainly focused on the promoter region within 2 kb upstream and the first intron region of the *Wx* gene but have rarely identified distal CREs (>2 kb from their nearest genes).

In this study, we combined the assay for transposase-accessible chromatin with high-throughput sequencing (ATAC-seq) data of developing seeds and bioinformatics analysis to identify CREs that regulate the expression of the key gene in starch biosynthesis, *Wx*, and obtained CRE mutants using CRISPR/Cas gene editing technology. Additionally, we analyzed the grain morphology, physicochemical quality traits, and agronomic traits of *Wx-cre* mutants and Nipponbare (NIP) near-isogenic lines with *Wx^a^* and *Wx^b^* alleles.

## 2 Method

### 2.1 Plant materials, transgene constructs, and rice transformation and growth conditions

**Figure 1:**
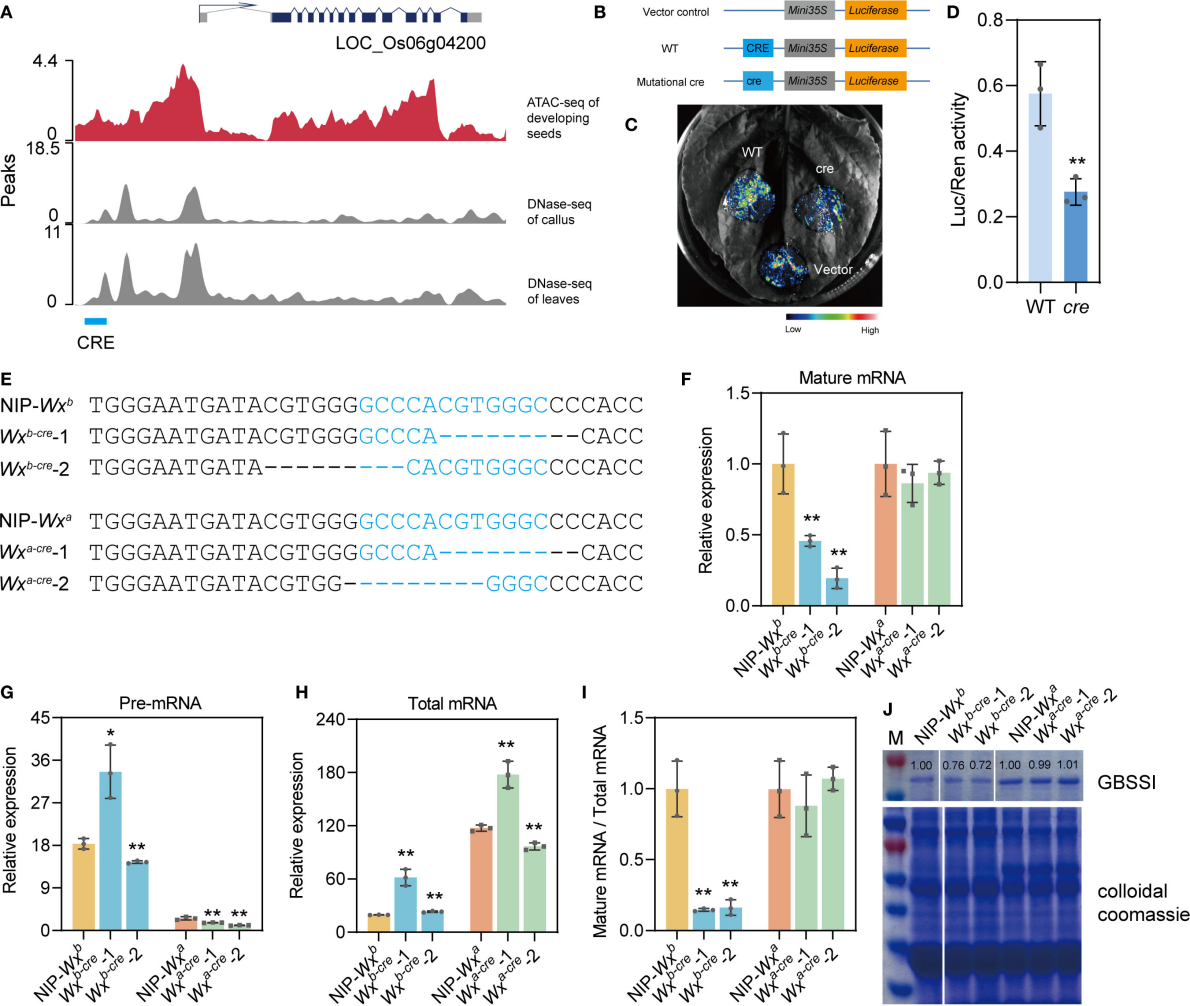
Mining and identification of cis-regulatory elements (CREs) related to the *Wx* gene. **(A)** Schematic diagram of predicted CREs of the *Wx* gene via ATAC-seq in developing seeds at 10 days after flowering (DAF), DNase-seq of leaves and callus (https://plantdhs.org/), and genome sequence analysis (http://rice.plantbiology.msu.edu/pub/data/). **(B–D)** Transient expression validation of screened CREs in the dual-luciferase reporter system. In detail, the tested sequence of WT CRE is 5′-GATACGTGGGGCCCACGTGGGCCCCACCATTT-3′, therein the underlined sequence is the predicted CRE; the tested sequence of mutational cre, 5′-GATACGTGGGCCCACCATT-3′, corresponds to a deletion of the predicted CRE. **(E)** Nucleotide variations of different homozygous mutants. The target CRE sequence is highlighted in blue font. “-” indicates a deletion. **(F–H)** Expression levels of mature *Wx* mRNA **(F)**, precursor *Wx* mRNA **(G)**, and total *Wx* mRNA **(H)** in developing seeds at 10 DAF of the wild-type and *cre* mutants. **(I)** Proportion of mature *Wx* mRNA of the total *Wx* mRNA. After normalization to *Actin* gene, the mean value of the control group was set to 1. **(J)** GBSSI protein level analyzed using SDS-PAGE. Rice total protein was used as the internal control. White lines represent the images from discontinuous lanes in a film. All data are presented as the mean ± SD. * and ** indicate significant differences from wild-type plants (*P < 0.05 and **P < 0.01) using two-tailed Student’s *t*-tests.

In this study, wild-type *Oryza sativa* L. ssp. *japonica* Nipponbare (NIP) was used for transformation, which carried the *Wx^b^* allele and thereby was named as NIP-*Wx^b^*. The near-isogenic line NIP-*Wx^a^* was derived from the *indica* cultivars GuiChao2 (GC2) through traditional rice hybridization and generated after at least six rounds of backcrossing with the recurrent parent NIP (Zhang et al., 2019). To detect the genetic backgrounds, NIP-*Wx^a^* was genotyped by whole-genome sequencing, as previously reported by our research group (Zhang et al., 2019). The gRNA target site was selected manually, according to the sequence information of CRE (**Figure 1**). To check for specificity, BLAST analyses of the gRNA target site were performed against the rice genome. The sgRNA expression cassette, including the CRE-targeting sequence, was constructed from SK-gRNA by PCR amplification with primers containing the gRNA sequence (**Supplementary Table S1**) and then subcloned into the CRISPR/Cas 9 vector by standard Golden Gate assembly (Engler et al., 2008). Rice calli from mature embryos were used as explants for *Agrobacterium*-mediated transformation, according to a previously published procedure (Liu et al., 1998).

Total genomic DNA was extracted from transformed rice leaves, as described previously (Yang et al., 2021). The targeted sequences were obtained using PCR with specific primers (**Supplementary Table S1**), and Sanger sequencing was performed to identify mutations in the target region of CRE. Three or more independent mutants were screened for further analysis, and all selected transgenic lines were homozygous.

All rice materials were planted in the paddy field of Yangzhou University (Yangzhou, Jiangsu Province, China) from April to October in 2021–2024 under safety supervision for genetically modified materials and the same climate and management conditions.

### 2.2 Transient assay verification in *Nicotiana benthamiana*

To examine the effects of predicted CREs, the wild-type CRE fragments with flanking sequences from the upstream region of the *Wx* gene (known as WT) and artificially mutated cre sequence (excluding CRE sequences, also known as cre) were each cloned upstream of the luciferase (*LUC*) coding region in an expression cassette driven by the *Mini35S* promoter in the pGreenII0800-LUC vector by using a dual-luciferase reporter system with two reporter genes, *LUC* and Renilla luciferase (*REN*) (Xing et al., 2020). The generated plasmids were transformed into *Agrobacterium tumefaciens* strain EHA105 for transient expression, as reported previously (Xiong et al., 2022). Briefly, *A. tumefaciens* strains with the constructed vector were resuspended in infection buffer (10 mM MgCl_2_, 10 mM MES, pH 5.6, and 200 μM acetosyringone) to an optical density at 600 nm (0.4) and infiltrated into 5-week-old *N. benthamiana* leaves. After incubation for 48 h, the LUC signal was visualized with a CCD system of Tanon 5200 Multi Imager (Tanon, Shanghai, China), and the infected leaves were harvested and used for measuring LUC/REN activities.

### 2.3 RT-PCR, qRT-PCR, and SDS-PAGE

Total RNA was extracted from young panicles of wild-type and mutated plants by using the Plant Total RNA Kit (TaKaRa, Beijing, China). The cDNAs were synthesized using Perfect Real Time PrimeScript RT reagent (TaKaRa). Real-time PCR was performed using TB Green^®^ Premix Ex Taq™ II (TaKaRa) and a LightCycler 480 system (Roche). The rice *Actin* gene was used as the internal reference to measure the relative expression levels of rice *Wx* and other 25 genes involved in starch synthesis. Gene-specific primers used for RT-PCR and qRT-PCR are listed in **Supplementary Table S1**.

For GBSSI activity and SDS-PAGE measurement, total protein was isolated from rice mature seeds, as described previously (Yang et al., 2021). The homogenate of total protein was then shaken for 30 min at room temperature and centrifuged at 10,000 ×g for 10 min. Then, the pellet was washed three times with the same buffer followed by twice with acetone and dried under vacuum. The dried pellet (50 mg) was then used for the extraction of granule-bound GBSSI according to Liu et al. (2014) and Zhang et al. (2019). SDS-PAGE was performed using standard procedures. Gels were stained with Coomassie Brilliant Blue R250 to examine the protein bands.

### 2.4 Agronomic trait analyses

The main agronomic traits, such as plant height, main panicle length, grains per panicle, effective tiller number, and seed setting rate, were investigated at maturity. The above samples have five biological replicates. At least 200 fully filled grains from panicles of a rice plant were used to measure 1,000-grain weight. The 1,000-grain weight data for each line were derived from the average of three panicles.

### 2.5 Grain physicochemical properties analyses

Harvested rice grains were dried in an oven at 37 °C for 1 week before analysis. Rice preparation and subsequent general quality measurements, such as apparent amylose content and rapid viscosity analyzer profile, were performed according to a previous study (Zhang et al., 2021). Differential scanning calorimetry (DSC) was performed using a DSC 200 F3 thermal analyzer (Netzsch Instruments NA LLC, Burlington, MA, USA) to analyze the gelatinization temperature of rice flour and isolated starches according to previous report (Zhang et al., 2013). The total starch content of the milled rice flour was analyzed using a total starch assay kit (K-TSTA, Megazyme, Ireland) according to the manufacturer’s assay procedure.

### 2.6 Statistical analysis

All data are presented as mean ± standard deviation (SD). Comparison of multiple transgenic and wild-type plants was performed using two-tailed Student’s *t*-tests. * and ** indicate statistical significance between transgenic and wild-type plants at *P* < 0.05 and *P* < 0.01, respectively.

## 3 Results and discussion

### 3.1 Identification of the distal CRE regulating *Wx* gene expression and the development of mutant varieties

In this study, we identified one distal CRE (occurring >2 kb upstream from the *Wx* gene) related to *Wx* by combining ATAC-seq data and bioinformatics analysis of developing seeds at 10 days after flowering (DAF) in *Oryza sativa* L. spp. *japonica* Nipponbare and comparative analysis of DNase I high-sensitivity sites obtained from published DNase-seq data of rice leaves and callus (https://plantdhs.org/) (**Figure 1A**). Therefore, this study focused on the distal CRE (5′-GCCCACGTGGGC-3′), which had not been reported in previous studies about *Wx* non-coding region editing (Huang et al., 2020; Zeng et al., 2020; Zhang et al., 2022). Preliminary assessment using the dual-fluorescence transient expression system showed that the CRE sequence enhanced the luciferase (LUC) activity in tobacco leaves compared with the plasmid control (**Figures 1B–D**).

In addition, we conducted sequence analysis using PlantCARE (https://bioinformatics.psb.ugent.be/webtools/plantcare/html/) and found that this CRE contains four motifs, i.e., G-box (CACGTG), ABRE2 (CCACGTGG), ABRE (ACGTG), and an unnamed motif (CGTGG). G-box is a ubiquitous element, which was identified in many various plant genes (Menkens et al., 1995). Multiple copies of ABREs (with a core ACGT) generally occur in the upstream of ABA/abiotic stress inducible genes (Hossain et al., 2010). These motifs are generally bound by the bZIP or bHLH transcription factors, playing a central regulatory role in plant growth and development, stress response, and signal transduction (Kong et al., 2018). Two predicted transcription factors (an MYB family transcription factor and an HD-ZIP III transcription factor) that can bind to this CRE sequence are being studied, which might help us to understand the regulation network of the *Wx* gene in rice.

To investigate the effect of *Wx*-CRE on AC in rice grain, we obtained *Wx*-CRE mutants using CRISPR/Cas9 technology. Transgenic plants were generated from two near-isogenic lines, NIP-*Wx^b^* and NIP-*Wx^a^*, via *Agrobacterium*-mediated transformation. Two homozygous *cre* mutant plants of NIP-*Wx^b^* or NIP-*Wx^a^* were selected for further analysis (**Figure 1E**). Sequencing analysis revealed that 7- and 3-bp deletions occurred in the CRE sequence of the *Wx^b^*-cre-1 and *Wx^b^*-cre-2 lines, respectively. *Wx^a^*-cre-1 had a 7-bp deletion, and *Wx^a^*-cre-2 had an 8-bp deletion in the CRE sequence (**Figure 1E**). The obtained *Wx* allelic variations have not been reported in previous studies on *Wx* non-coding region editing.

We then evaluated GBSSI mRNA and protein levels in developing seeds at 10 DAF and mature seeds of mutants and the wild type (WT). In the *Wx^a^* background, the expression levels of GBSSI mRNA and protein were almost unchanged compared with WT (**Figures 1F, J**). *Wx^b^-cre* mutants expressed lower mRNA and protein levels than WT (**Figures 1F, J**).

In the developing endosperm, the *Wx* gene had two mRNA transcripts, namely, a 3.3-kb precursor mRNA (pre-mRNA) and a 2.3-kb mature mRNA (mature-mRNA, which is translated into the Wx protein). The endosperm AC and Wx protein content were significantly correlated with the mature mRNA content and the ability to excise intron I from the leader sequence of the *Wx* transcript (Wang et al., 1995). Thus, the relative amounts of *Wx* gene pre-mRNA, mature mRNA, and total mRNA (including the 3.3-kb precursor mRNA and 2.3-kb mature mRNA) in developing seeds of different mutant and WT plants at 10 DAF were detected using quantitative real-time PCR with specific primers (**Supplementary Figure S1**, **Supplementary Table S1**). As expected, the relative expression level of mature mRNA was reduced in the *Wx^b^*-*cre* mutants compared with WT plants that carried the *Wx^b^* allele, but similar results were not observed in *Wx^a^-cre* mutants (**Figure 1F**). The relative expression levels of total mRNA and pre-mRNA of the *Wx* gene exhibited inconsistent changes between the mutant and the WT no matter in the *Wx^a^* or *Wx^b^* background (**Figures 1G, H**). The proportion of mature *Wx* mRNA to total *Wx* mRNA significantly decreased in the mutants from the *Wx^b^* background after normalization, but no change was observed in the *Wx^a^*-*cre* mutant plants (**Figure 1I**). However, the total *Wx* mRNA showed a higher level in *Wx^b^*-*cre* mutants than that of WT (**Figure 1H**). The likely reason is that CRE editing may alters the chromatin conformation, in turn affecting the efficiency of transcription machinery (Rippe and Papantonis, 2025). These results suggest that this CRE regulates the transcription and protein levels of the *Wx* gene by influencing transcriptional splicing, thereby fine-tuning AC in rice via the *Wx^b^* allele.

### 3.2 Physicochemical property analysis of mutant grains

**Figure 2:**
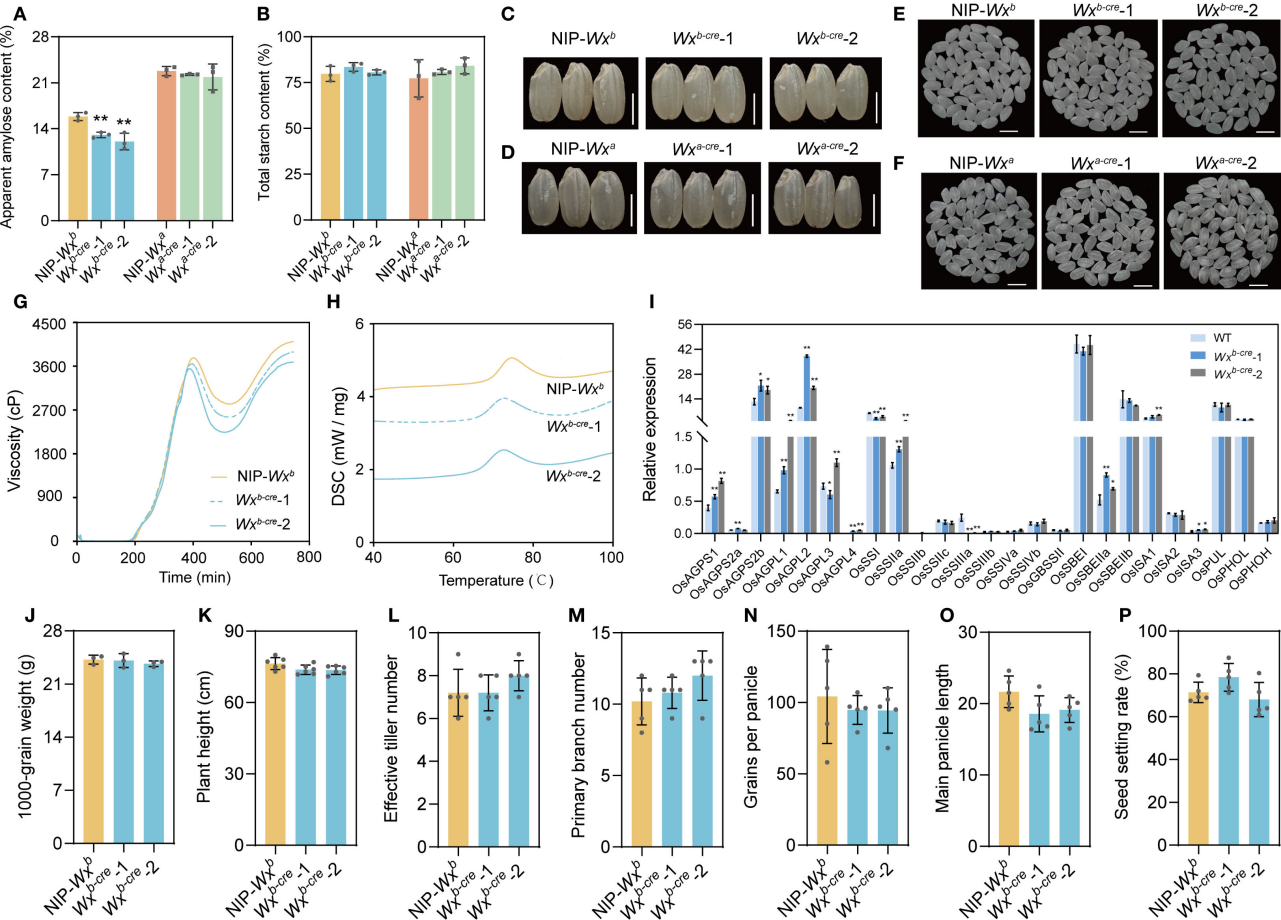
Effects of *Wx^b^* and *Wx^a^* allele backgrounds on *cre* mutants. **(A, B)** Contents of amylose **(A)** and total starch **(B)** in rice flour of mutants and WT. **(C)** Rice grain morphology of WT and *Wx^b^-cre* mutants with a *Wx^b^* allele. Bar, 3 mm. **(D)** Rice grain morphology of WT and *Wx^a^-cre* mutants with a *Wx^a^* allele. Bar, 3 mm. **(E)** Polished rice appearance of WT and *Wx^b^-cre* mutants with a *Wx^b^* allele. Bar, 5 mm. **(F)** Polished rice appearance of WT and *Wx^a^-cre* mutants with a *Wx^a^* allele. Bar, 5 mm. **(G)** Rapid viscosity profiles of rice flour. **(H)** Thermal properties of rice flour. **(I)** Expression levels of genes involved in starch biosynthesis in *Wx^b^-cre* mutants. The reference gene was *Actin*. Each gene name is indicated by a simplified representation. **(J–P)** Comparative analysis of main grain agronomic traits between *Wx^b^-cre* mutants and WT in rice with a *Wx^b^* allele. All data are presented as the mean ± SD. * and ** indicate significant differences from wild-type plants (*P < 0.05 and **P < 0.01) using two-tailed Student’s *t-*tests.

AC was analyzed to confirm the effect of CRE on different backgrounds (*Wx^a^* or *Wx^b^*). The apparent AC in the rice flour of the *Wx^b^-cre* mutant decreased by 21.43%–31.51% compared with WT with the *Wx^b^* allele (**Figure 2A**), but there was no significant difference in the total starch content between the mutants and WT (**Figure 2B**). There were no significant differences in the AC and total starch content between *Wx^a^*-cre mutants and WT plants, which carried the *Wx^a^* allele (**Figures 2A, B**).

AC is closely linked to grain appearance quality and physicochemical properties in rice (Tian et al., 2009). Therefore, we evaluated the grain appearance quality and physicochemical properties of mutant lines and their corresponding WTs. The mutants exhibited a normal seed morphology, chalkiness rate, and chalkiness degree compared with WT (**Figures 2C–F**, **Supplementary Figure S2**). In the rice mutant with the *Wx^b^* background, the rapid viscosity analysis indicated that the decrease in AC caused the slightly lower final viscosity, suggesting an increased gelatinous consistency (**Figure 2G**). Compared with grains of WT plants, those of mutant plants had a lower gelatinization temperature in *Wx^b^*-*cre* lines (**Figure 2H**, **Supplementary Table S2**). These results indicate that the reduction in AC results in a softer texture in mutant rice compared with WT-*Wx^b^*.

### 3.3 Main agronomic traits were not affected in mutants

We assessed whether agronomic traits were affected in the *cre* mutant plants. The *cre* mutant lines and WT plants grew normally during the growing season. We investigated the main agronomic traits of the mutant lines and WT after maturation, and there were no statistically significant differences in plant height, main panicle length, or effective tiller number between the *cre* mutant and WT plants (**Figures 2J–P**). In addition, the rice grain yield traits, namely, grain number per panicle, seed setting rate grain size, and 1,000-grain weight, were similar in the mutants and the corresponding WT plants (**Figures 2J–P**). These findings indicate that editing the CRE(s) of *Wx* can fine-tune AC without affecting other agronomic traits.

### 3.4 CRE mutant causes the transcriptional reprogramming of starch metabolism

To determine whether changes to the *Wx* expression level also affected starch metabolism, we compared the expression profiles of 25 genes involved in starch biosynthesis, representing five classes of enzymes (AGPase, SS, SBE, DBE, and Pho), in the seeds at 10 DAF between *Wx^b^-cre* mutants and WT.

A significant increase in the expression of AGPase genes was observed in *Wx^b^-cre* mutants compared with WT. *OsAGPS2b* and *OsAGPL2* were strongly expressed in the seeds and were significantly upregulated in the *Wx^b^-cre* lines compared with WT (**Figure 2I**). This might be because ADP-glucose accumulation within cells induces AGPase activity (Pérez et al., 2019). *OsSSI*, *OsSSIIIa*, and *OsSSIIIa* were significantly downregulated in developing seeds of *Wx^b^-cre*-1 and *Wx^b^-cre*-1 lines, but *OsSSIIa* and *OsSSIIb* were upregulated in these mutants (**Figure 2I**). These results suggest that transcriptional reprogramming of genes encoding starch synthases resulted in the regulation of the *Wx* gene. For starch branched enzyme genes, only the expression of *SBEI* showed a slight increase in the mutants (**Figure 2I**). In the *Wx^b^-cre* mutants, *OsISA1* and *OsISA3* were significantly upregulated, but no significant changes were observed in *OsPUL*, *OsPHOL*, or *OsPHOH* (**Figure 2I**). The changes in genes such as SSs, SBEs, DBEs, and Phos suggested that the amylopectin properties have been fine tuned. The above results indicated that starch biosynthesis metabolism is a complex regulatory network that may be regulated by common transcription factors or transcription factor complexes (Huang et al., 2021).

In summary, we present a novel and effective strategy for decreasing AC in rice inbred lines carrying a *Wx^b^* allele. This study shows that CREs have different effects depending on the *Wx* allele background. The results of this study provide invaluable insights into the regulation of *Wx* gene expression and a strategy for improving the cooking and tasting quality using varieties with different *Wx* alleles.

## Data Availability

The original contributions presented in the study are included in the article/**Supplementary Material**. Further inquiries can be directed to the corresponding authors.
