# Supplementary material for: A distal regulatory element regulates Wx gene expression and the amylose content in rice
Source: Front Plant Sci. 2025 Oct 8;16:1694305. doi: 10.3389/fpls.2025.1694305 (PMC12540380; doi:10.3389/fpls.2025.1694305)
Supplement: Supplementary file 1 [file DataSheet1.pdf]

SUPPLEMENTAL INFORMATION

Supplementary Figures

Supplementary Figure 1. Localization of *Wx* gene primers for expression analysis of different transcripts.

Supplementary Figure 2. Comparative analysis of chalkiness rate and chalkiness degree between Wx^b-*cre* mutants and WT plants.

Supplementary Tables

Supplementary Table 1. List of primers used in this study

Supplementary Table 2. Thermal properties of rice flour from different mutants and WT lines under Wx^b background

Supplementary Data

Supplementary Figures

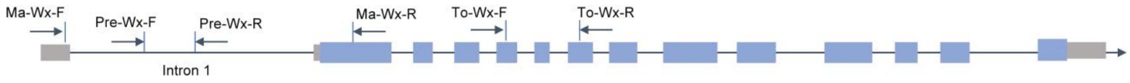

Supplementary Figure 1. Localization of *Wx* gene primers for expression analysis of different transcripts.

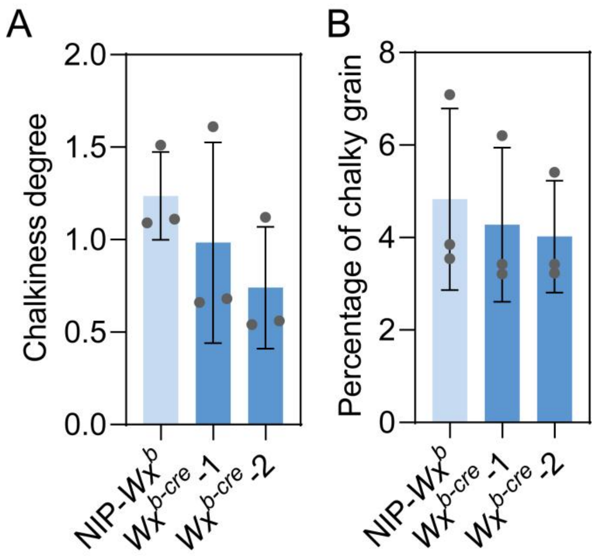

Supplementary Figure 2. Comparative analysis of chalkiness rate and chalkiness degree between $Wx^{b\text{-}cre}$ mutants and WT plants.

Supplementary Tables

Supplementary Table 1. List of primers used in this study

Primer name	Forward primer (5′–3′)	Reverse primer (5′–3′)
maWx	ATTCCTTCAGTTCTTTGTCTATCTCA	ATGGTGGTTGTCTAGCTGTTGC
prWx	ATCTTTCATTGCTCGTTTTTCCTTA	GCCTAACCAAACATAACGAACGA
toWx	CGTCATTCCTGGAGAAGGTTTG	CAGACGAACACAACATCCTCACC
CRE-gRNA	ATGATACGTGGGGCCCACGTGGG	
Wx1p	GGAGAAGAGATGTGAGAGAGAGTTG	CCGTCCTTCAACCGCAAA
Wx-mLUC	gtcgacggtatcgataagcttTGCCACCGTCGCAGGAGAA	cgctctagaactagtggatccTCCGTCCTTCAACCGCAAA
OsAGPS1-q	GTGCCACTTAAAGGCACCATT	CCCACATTTCAGACACGGTTT
OsAGPS2a-q	ACTCCAAGAGCTCGCAGACC	GCCTGTAGTTGGCACCCAGA
OsAGPS2b-q	AACAATCGAAGCGCGAGAAA	GCCTGTAGTTGGCACCCAGA
OsAGPL1-q	GGAAGACGGATGATCGAGAAAG	CACATGAGATGCACCAACGA
OsAGPL2-qF	AGTTCGATTCAAGACGGATAGC	CGACTTCCACAGGCAGCTTATT
OsAGPL3-qF	AAGCCAGCCATGACCATTTG	CACACGGTAGATTCACGAGACAA
OsAGPL4-qF	TCAACGTCGATGCAGCAAAT	ATCCCTCAGTTCCTAGCCTCATT
OsSSI-qF	GGGCCTTCATGGATCAACC	CCGCTTCAAGCATCCTCATC
OsSSIIa-q	GCTTCCGGTTTGTGTGTTCA	CTTAATACTCCCTCAACTCCACCAT
OsSSIIb-q	TAGGAGCAACGGTGGAAGTGA	GTGAACGTGAGTACGTGACCAAT
OsSSIIc-q	GACCGAAATGCCTTTTTCTCG	GGGCTTGGAGCCTCTCCTTA
OsSSIIIa-q	GCCTGCCCTGGACTACATTG	GCAAACATATGTACACGGTTCTGG
OsSSIIIb-q	ATTCCGCTCGCAAGAACTGA	CAACCGCAGGATAACGGAAA
OsSSIVa-q	GGGAGCGGCTCAAACATAAA	CCGTGCACTGACTGCAAAAT
OsSSIVb-q	ATGCAGGAAGCCGAGATGTT	ACGACAATGGGTGCCAAGAT
OsGBSSII-q	AGGCATCGAGGGTGAGGAG	CCATCTGGCCCACATCTCTA
OsSBEI-q	TGGCCATGGAAGAGTTGGC	CAGAAGCAACTGCTCCACC
OsSBEIIa-q	GCCAATGCCAGGAAGATGA	GCGCAACATAGGATGGGTTT
OsISA1-q	TGCTCAGCTACTCCTCCATCATC	AGGACCGCACAACTTCAACATA
OsISA2-q	TAGAGGTCCTCTTGGAGG	AATCAGCTTCTGAGTCACCG
OsISA3-q	ACAGCTTGAGACACTGGGTTGAG	GCATCAAGAGGACAACCATCTG
OsPUL-q	ACCTTTCTTCCATGCTGG	CAAAGGTCTGAAAGATGGG
OsPHOL-q	TTGGCAGGAAGGTTTCGCT	CGAAGCCTGAAGTGAACTTGCT
OsPHOH-q	CACCAAGACGAAGCTCATCAAG	TTCACTCGTTGCTGGGTTCTC

Supplementary Table 2. Thermal properties of rice flour from different mutants and WT lines under Wxb background

Lines	DSC parameters			
	To (°C)	Tp (°C)	Tc (°C)	ΔH (J G^{-1})
NIP-*Wx^b*	68.73±0.25	74.23±0.21	82.60±0.10	6.60±0.33
$Wx^{b\text{-}cre}$-1	66.27±0.06**	72.70±0.20**	82.00±0.20**	6.56±0.27
$Wx^{b\text{-}cre}$-2	65.87±0.71**	72.80±0.26**	81.47±0.58*	6.16±0.59

* and ** indicate significant differences from wild-type plants (*$P < 0.05$ and **$P < 0.01$) by using two-tailed Student's *t*-tests.
